# A Mobile-Based Mindfulness Intervention for Reducing Demoralization in Patients on Maintenance Hemodialysis: Randomized Controlled Trial

**DOI:** 10.2196/90324

**Published:** 2026-08-07

**Authors:** Xingxing Shen, Huanhuan Li, Fang Sun, Meihong Gu, Hui Zhang, Fenfen Zhu

**Affiliations:** 1 Department of Psychology, Renmin University of China, Floor 10, Suite D, Huixian Building, 59 Zhongguancun Street, Haidian District, Beijing, 100872, China, 86 13439892584; 2 School of Nursing, Suzhou Vocational Health College, Suzhou, Jiangsu, China; 3 Research Center for Social Psychological Science and Engineering, Renmin University of China, Beijing, China; 4 School of Humanities and Social Sciences, Harbin Engineering University, Harbin, Heilongjiang, China; 5 Blood Purification Center, Jiangyin People's Hospital, Jiangyin, Jiangsu, China

**Keywords:** mobile-based mindfulness intervention, maintenance hemodialysis, demoralization, symptom burden, randomized controlled trial

## Abstract

**Background:**

Patients with end-stage renal disease receiving maintenance hemodialysis experience a substantial symptom burden, and demoralization syndrome—characterized by helplessness, hopelessness, and loss of meaning—is highly prevalent and strongly associated with impaired quality of life. However, the psychological mechanisms linking symptom burden to demoralization remain poorly understood, and scalable, evidence-based interventions targeting demoralization in this population are limited.

**Objective:**

This study aimed to (1) examine the mechanisms linking symptom burden to demoralization through coping strategies and trait mindfulness and (2) evaluate the efficacy of a mobile-based mindfulness intervention (MMI) in reducing demoralization.

**Methods:**

Using an explanatory sequential mixed methods design, study 1 involved a cross-sectional survey (N=305) and semistructured interviews (n=14) among patients receiving hemodialysis to identify psychological pathways and clinical intervention needs. Study 2 was a randomized controlled trial in which 72 patients with moderate-to-severe demoralization (Demoralization Scale-II ≥10) were randomized (1:1) to a 6-week WeChat-delivered MMI (n=36) or usual care (n=36). Quantitative data were analyzed using moderated mediation and repeated-measures ANOVA, and qualitative data were analyzed via content analysis.

**Results:**

Study 1 quantitative results showed that trait mindfulness negatively moderated the paths from the symptom burden to demoralization (β=−0.11, 95% CI −0.17 to −0.05) and from avoidance coping to demoralization (β=−0.07, 95% CI −0.12 to −0.01). Qualitatively, 4 themes emerged: the psychological experience of demoralization, barriers to the alleviation of demoralization, facilitators of demoralization alleviation, and a strong preference for dialysis-adapted, flexible psychological support. In the study 2 randomized controlled trial, compared with the control group, the MMI group showed significant postintervention improvements in demoralization (mean difference [MD] −5.06, 95% CI −6.92 to −3.20), symptom burden (MD −29.15, 95% CI −46.93 to −11.37), and trait mindfulness (MD 6.48, 95% CI 4.65 to 8.31).

**Conclusions:**

Avoidance coping represents an important pathway associated with demoralization among patients with end-stage renal disease receiving maintenance hemodialysis, and trait mindfulness may serve as a protective psychological resource. A tailored MMI showed promising effects in reducing demoralization and improving related psychological outcomes. These findings highlight the potential of scalable digital mindfulness approaches to support psychological care in routine hemodialysis management.

## Introduction

Hemodialysis is a life-sustaining but highly burdensome treatment for patients with end-stage renal disease [1]. Despite advances in dialysis technology and clinical management, many patients undergoing chronic hemodialysis experience persistent psychological distress due to the long-term nature of the treatment and related complications [2,3]. Demoralization, characterized by loss of meaning, hopelessness, and impaired coping [4], is increasingly recognized in nephrology, with 59.4% of patients receiving hemodialysis exceeding the clinical cutoff in a multicenter study [5]. Heightened demoralization has been strongly and consistently linked to adverse psychological outcomes, poorer treatment adherence, and diminished quality of life among these patients [6]. An understanding of its mechanisms and the development of effective interventions are thus critical for improving patients’ mental health and quality of life.

A growing body of evidence suggests that the symptom burden is a central pathway through which hemodialysis exerts its psychological toll. Defined as the cumulative incidence, frequency, and severity of disease-related symptoms and the associated distress [7], the symptom burden has emerged as one of the most robust predictors of emotional dysfunction in chronically ill populations [8]. In patients receiving hemodialysis, high levels of fatigue, pain, sleep disturbance, and autonomic discomfort create persistent physiological strain that can overwhelm psychological resilience. Greater symptom burdens have been significantly associated with elevated demoralization [9,10], but the mechanisms linking these 2 constructs remain insufficiently understood, limiting the development of targeted clinical strategies.

According to the stress and coping theory [11], individuals appraise illness-related stressors not only based on their objective severity but also in terms of their coping styles. Thus, coping styles may regulate cognition regarding physiological symptoms, resulting in different health outcomes [12]. Among patients, these styles are commonly classified as centered on confrontation, avoidance, and resignation [13]. As mediators, coping styles link stressors to health outcomes [14-16]. Positive coping (eg, confrontation) buffers demoralization [17], whereas avoidance and resignation predict greater distress and demoralization [18]. Prior research has shown that symptom burden increases demoralization through resignation coping among patients with cancer [19]. However, this focus on resignation leaves unresolved whether different coping strategies exert differential effects along the symptom burden-demoralization pathway in patients receiving hemodialysis. Moreover, whether this indirect pathway is moderated by other potential protective factors remains unexplored. Identifying such moderators is particularly valuable for guiding the development of targeted and scalable intervention strategies.

In this context, trait mindfulness, characterized by nonjudgmental awareness and acceptance of present experiences, has been identified as a protective factor against stress and emotional dysregulation [20]. It is positively associated with adaptive behaviors and health outcomes [21,22] and negatively associated with anxiety and negative affect [20]. Emerging evidence suggests that mindfulness buffers the adverse effects of stress on emotional and behavioral functioning [23,24]. Despite these well-documented benefits, its role in mitigating demoralization, particularly among patients receiving hemodialysis, has rarely been examined in a systematic manner.

Importantly, trait mindfulness is not only a dispositional characteristic but also a modifiable psychological capacity, making it a promising target for intervention. Mindfulness-based interventions (MBIs) have demonstrated efficacy in the reduction of psychological distress and emotional dysregulation across a range of chronic illnesses [25-27]. A systematic review and meta-analysis of 47 randomized controlled trials (RCTs) found moderate evidence that mindfulness meditation programs improve anxiety, depression, and pain in adult clinical populations [28]. Traditional MBIs, such as mindfulness-based stress reduction, typically involve face-to-face group sessions led by trained instructors [29]. Although effective, these formats pose practical challenges for patients receiving hemodialysis, whose treatment schedules, physical limitations, and fatigue often hinder regular participation [30].

Mobile-based MBIs have emerged as a promising alternative by improving accessibility, flexibility, and scalability while reducing implementation costs [31]. Growing evidence from RCTs suggests that these interventions effectively reduce anxiety and depression among individuals experiencing emotional distress, potentially through improvements in cognitive mechanisms [32,33]. To our knowledge, no published RCT has evaluated a mobile-based mindfulness intervention (MMI) specifically targeting demoralization in patients receiving hemodialysis.

To address this gap, this study was guided by 2 complementary theoretical frameworks: the stress system model [34] and the Intention-Attention-Attitude model [35]. The former conceptualizes demoralization as a stress response to symptom burden, whereas the latter informed the intervention design by targeting intention through psychoeducation, attention through daily audio-guided practice, and attitude through self-compassion exercises tailored to patients receiving hemodialysis. Based on these frameworks, a 6-week MMI with weekly thematic modules was developed. Thus, this study evaluated the effectiveness of MMI for patients receiving hemodialysis and examined the psychological mechanisms associated with demoralization. We hypothesized that compared with the control group, participants receiving the intervention would show increased trait mindfulness and reduced demoralization after 6 weeks.

## Methods

### Study 1: Mechanisms of Demoralization

#### Study Design

This study used a sequential mixed methods design conducted in 2 phases. Phase 1 used a cross-sectional survey to examine the associations among symptom burden, coping styles, trait mindfulness, and demoralization in patients receiving hemodialysis. Phase 2 comprised a qualitative study using semistructured interviews to explore the factors influencing demoralization, as well as patients’ intervention needs.

#### Quantitative Phase: Participants and Survey Procedure

Convenience sampling was used to recruit patients with end-stage renal disease who were receiving hemodialysis at the hemodialysis center of a tertiary-grade hospital in Jiangsu Province, China, between December 2023 and February 2024.

The inclusion criteria were (1) aged 18 to 65 years, (2) receiving maintenance hemodialysis at least 3 times weekly for 3 or more months, and (3) able and willing to participate. The exclusion criteria were (1) major psychiatric disorders or severe cognitive impairment, (2) serious complications or unstable physical conditions, and (3) the receipt of other psychological interventions or pharmacological treatments.

Data were collected through an online survey platform. Participants were identified using hospital medical record IDs to prevent duplicate enrollment. After excluding 35 incomplete questionnaires, 305 participants were included in the final analysis (male: n=181, 59.34%; aged 61‐65 y: n=88, 28.85%; and with education levels below junior high school: n=223, 73.11%).

#### Qualitative Phase: Participants and Interview Procedure

Participants were purposively selected from survey respondents with Demoralization Scale-II (DS-II) scores ≥10, using maximum variation sampling (age, education, occupation, residence, primary disease, and comorbidity burden). Recruitment continued until thematic saturation was reached, with no new codes emerging after the 12th interview and 2 additional interviews confirming saturation, yielding a final sample of 14 participants (8 male and 6 female participants).

The interview outline is provided in Section S1-1 in Multimedia Appendix 1. Semistructured interviews (30‐60 min) were conducted via Tencent Meeting (Tencent Holdings Ltd) on nondialysis days. With participants’ consent, interviews were video-recorded, transcribed within 24 hours, manually verified, anonymized, and imported into NVivo 12.0 (QSR International Pty Ltd) for analysis.

#### Measures

##### Sociodemographic and Clinical Characteristics

Age, sex, education, occupational status, marital status, religious beliefs, residence, medical payment method, family disease burden, self-care status, primary kidney disease, dialysis duration, and the number of long-term medications (a proxy for comorbidity burden) were collected.

##### DS-II

The DS-II was used to assess demoralization over the previous 2 weeks [36] using a 3-point Likert scale (0=never, 1=sometimes, and 2=often). Total scores range from 0 to 32, with scores ≥10 indicating moderate-to-severe demoralization. In this study, the Cronbach α was 0.907.

##### Unidimensional Mindful Attention Awareness Scale

The Mindful Attention Awareness Scale was used to measure trait mindfulness [21] using a 6-point Likert scale. The total scores range from 15 to 90 (≥66=high, 41‐65=moderate, ≤40=low mindfulness level). In this study, the Cronbach α was 0.954.

##### Chinese Version of the Medical Coping Modes Questionnaire

The Medical Coping Modes Questionnaire was used to assess coping strategies in response to their illness, including 20 items across 3 dimensions (confrontation, avoidance, and resignation) [37]. Rated on a 4-point Likert scale, higher dimension scores indicate a greater tendency to use the corresponding coping strategy. In this study, the Cronbach α values for the 3 dimensions were 0.673, 0.747, and 0.786, respectively. The relatively lower α for confrontation is comparable to that reported in the original Chinese validation study (α=0.69) [37].

##### Dialysis Frequency, Severity, and Symptom Burden Index

Dialysis Frequency, Severity, and Symptom Burden Index was used to assess the severity of symptoms in patients undergoing hemodialysis over the preceding 7 days [38]. Symptoms were recorded as present or absent, with higher scores indicating greater symptom burden. In this study, the Cronbach α was 0.957.

### Statistical Analysis (Study 1)

Quantitative analyses were performed using IBM SPSS 26.0 and PROCESS macro (version 4.2; The Guilford Press). Demographic and clinical characteristics that differed significantly between groups were entered as covariates. Following correlational and multiple regression analyses, moderated mediation was tested using the ordinary least square–based path analysis [39], with 5000 bootstrap resamples after mean-centering continuous variables. Effects were considered significant when the 95% CI excluded 0. Significant interactions were probed using simple slope analyses [40].

Interview data were analyzed using traditional content analysis [41] with NVivo (version 12; Lumivero). Two researchers (a master’s student in psychology and a clinical nurse) independently coded the first 3 transcripts to develop an initial codebook, which was iteratively refined throughout the analysis. To enhance trustworthiness, preliminary themes were reviewed by a psychology professor and a chief nephrologist, and thematic summaries were returned to 3 participants for confirmation.

### Study 2: Effects of MMI

#### Study Design

To evaluate the efficacy of a 6-week MMI, a 2 (time: baseline, postintervention) × 2 (group: intervention, control) RCT design was conducted. Participants were recruited between February 10 and March 7, 2024, and completed follow-up between March 8 and April 19, 2024.

#### Participants

Eligible participants were selected from study 1 (patients receiving hemodialysis with a DS-II score≥10). Using an online random number generator [42], an independent researcher randomly selected 72 eligible participants, who all agreed to participate (100% consent rate). They were subsequently randomized and allocated in a 1:1 ratio to the intervention or control group (36 per group). Owing to the nature of the intervention, participants and interventionists were not blinded; however, outcome assessors and data analysts remained blinded.

#### Intervention

The MMI intervention was based on the Intention-Attention-Attitude model [35] and was informed by the qualitative findings from study 1. The intervention protocol was finalized under the guidance of 5 experts (Section S1-2 in Multimedia Appendix 1).

**Figure 1.**
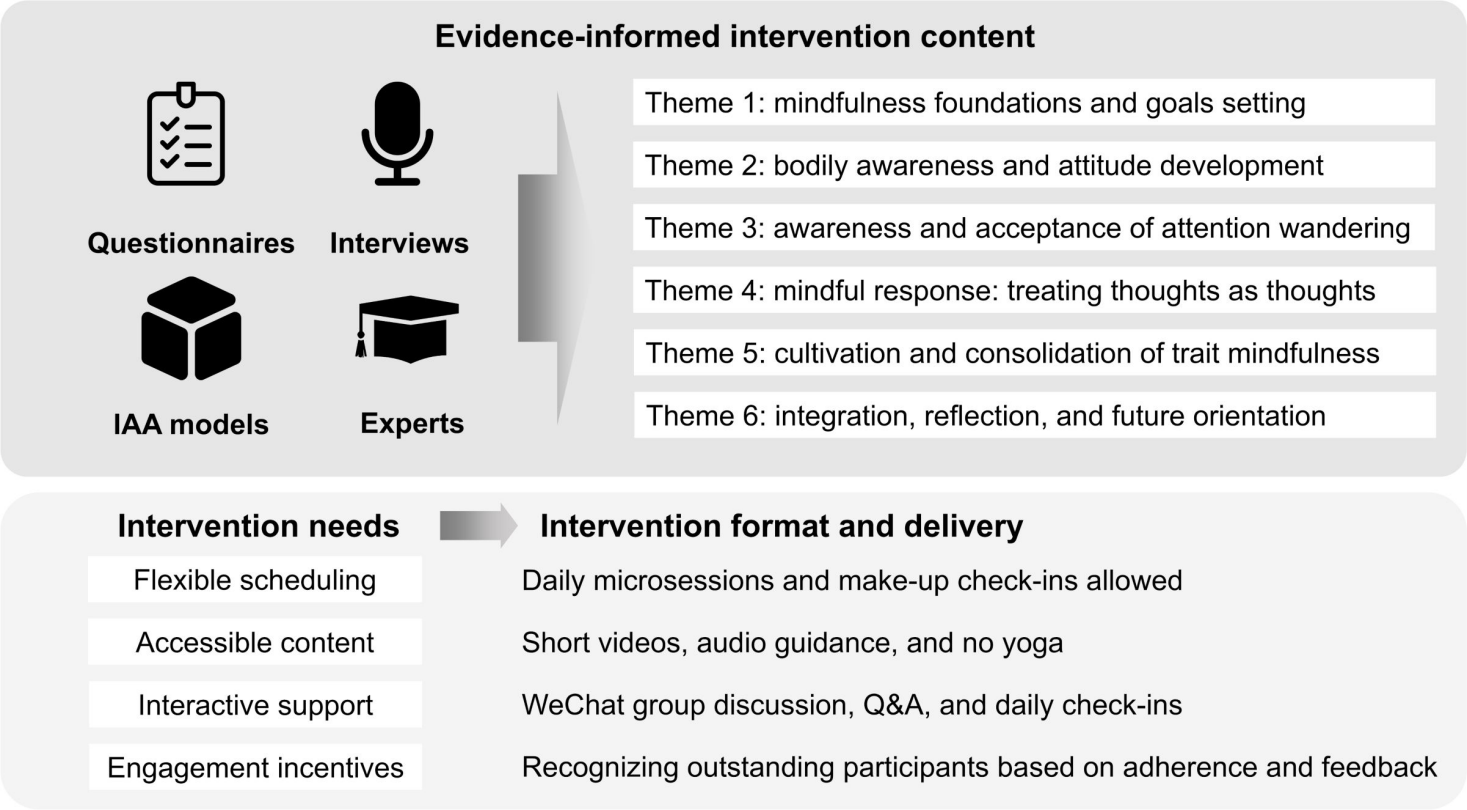
Development framework of the mobile-based mindfulness intervention (MMI) intervention, illustrating how the intervention content and delivery format were informed. IAA: Intention‑Attention‑Attitude.

To improve feasibility for patients receiving hemodialysis, the standard 8-week mindfulness program was adapted into a 6-week mobile-based intervention with dialysis-specific modifications (eg, replacing yoga with adapted mindfulness exercises and incorporating self-compassion prompts during body scans). Weekly modules focused on mindfulness foundations, body awareness, acceptance, and mindful coping (Figure 1). For example, during body scans, participants were guided to focus on key areas such as the kidneys and arteriovenous fistula, while self-compassion prompts were incorporated (eg, “When scanning the left forearm with the fistula, you may say to it: You are my lifeline. Thank you for accompanying me and sustaining my life. Thank you”) to enhance symptom awareness and acceptance. Repeated engagement was expected to strengthen momentary mindfulness skills and gradually translate into improvements in trait mindfulness over the 6-week intervention period.

The intervention was delivered through the WeChat E-Check-in Mini Program (version 2.7.0; Shenzhen Xiao’e Network Technology Co, Ltd). Participants completed one 10- to 15-minute session daily, including psychoeducation, audio-guided mindfulness practice, and reflective exercises. Automated reminders, daily check-ins (with up to 2 make-up sessions per week), and a WeChat support group were provided throughout the intervention. The group was moderated by a psychology-trained research coordinator who provided responses to content- or technology-related inquiries within 24 hours and facilitated peer discussion.

Participants in the control group received usual care through a separate WeChat group, including twice-weekly health education and daily self-management tips. Separate groups were used to minimize contamination, and no apparent contamination was identified during the study.

#### Outcomes

Assessments were completed online at baseline and immediately post intervention. The primary outcome was demoralization, and secondary outcomes were trait mindfulness, coping styles, and symptom burden. The assessment tools of outcome measures were consistent with those used in study 1. Adherence was defined as the completion of the daily miniprogram check-in during the 42-day intervention, based on back-end records.

#### Statistical Analysis (Study 2)

Analyses were performed using IBM SPSS 26.0 following the intention-to-treat principle. Baseline characteristics were compared using independent-samples *t* tests and chi-square tests. Missing data were handled using multiple imputation (5 datasets).

Intervention effects were evaluated using repeated-measures ANOVA. When the sphericity assumption was violated, Greenhouse-Geisser corrections were applied, and Bonferroni-adjusted post hoc comparisons were performed. Per-protocol analyses were conducted as sensitivity analyses among participants meeting the predefined adherence criterion.

### Ethical Considerations

Both studies were approved by the Ethics Committee of the Department of Psychology at Renmin University of China (IRB 23‐082; December 15, 2023) and by Jiangyin People’s Hospital (2024 Lun Shen Yan 098; May 10, 2024) and were registered with the Chinese Clinical Trial Registry (ChiCTR2500107906; August 20, 2025). Although ethical approval was obtained before recruitment, the trial was registered retrospectively. The registration application was submitted on December 21, 2023, before participant enrollment. However, completion was delayed due to the mandatory administrative review process required for clinical trial registration in China, including prior filing and institutional reviews within the National Medical Research Registry System of the National Health Commission.

Written informed consent was obtained from all participants (and legal guardians, where applicable). All data were anonymized using unique identifiers, encrypted, and stored on secure servers for 5 years after study completion. Participants were able to withdraw at any time without penalty, received free access to the mindfulness program, and received modest financial compensation after completing the assessments.

## Results

### Study 1: Examination of the Mechanisms of Demoralization

#### Sample Characteristics and Variable Correlations

**Figure 2.**
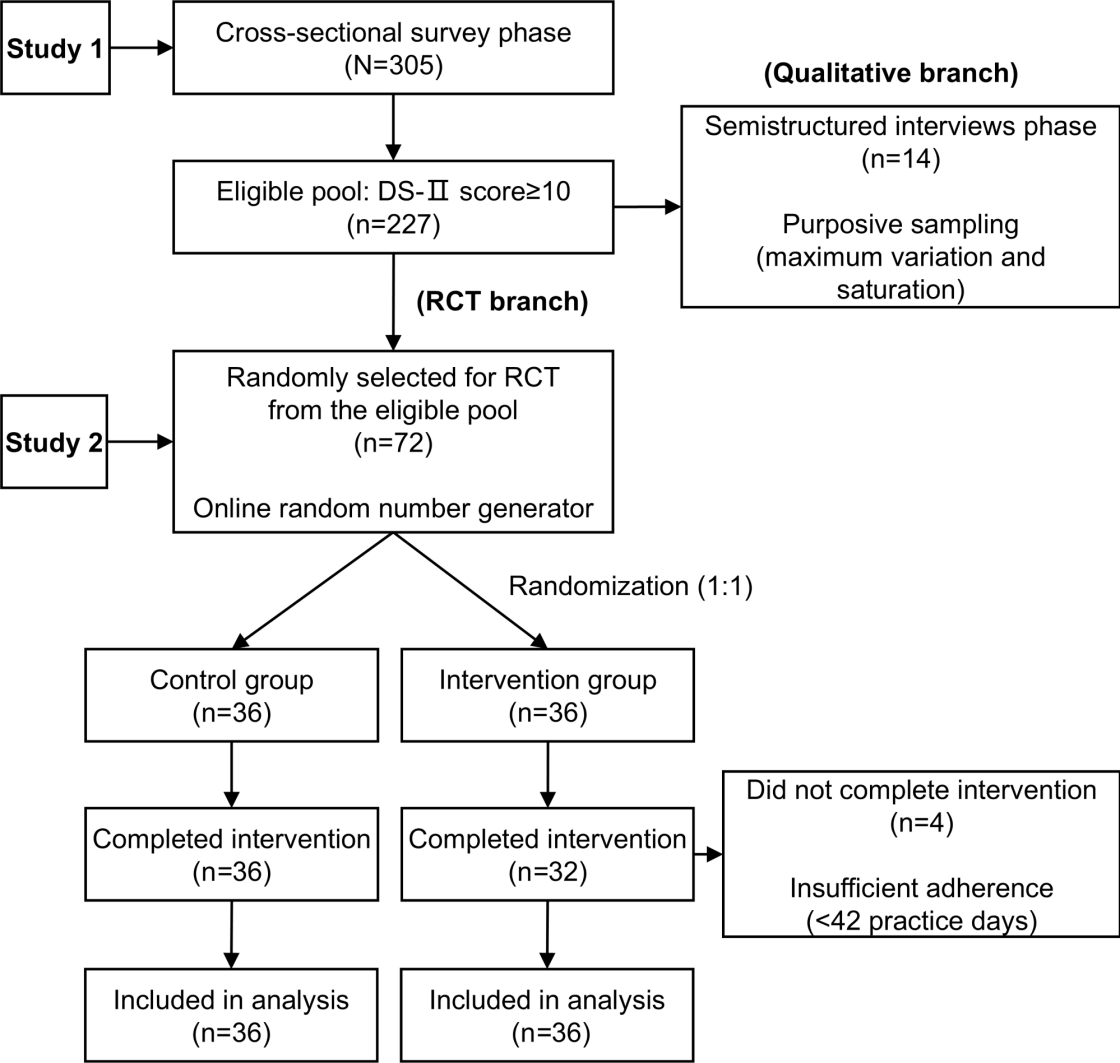
Overall study flowchart and participant progress through the qualitative and randomized controlled trial (RCT) phases (analyzed by intention-to-treat). DS-II: Demoralization Scale-II.

Eligible participants were selected from study 1 (Figure 2). The participants’ demographic and clinical characteristics are detailed in Table 1. Demoralization scores differed significantly across participants’ age, education level, occupational status, religious beliefs, place of residence, self-care status, primary disease, and the type of long-term medication. These variables were controlled for in subsequent analyses.

The demoralization scores correlated negatively with trait mindfulness and confrontation coping scores and positively with avoidance coping, resignation coping, and symptom burden scores (Table 2).

**Table 1.** Baseline characteristics of patients (N=305).

Variables	n (%)	Demoralization score, mean (SD)	*t* or *F* (*df*)	*P* value
Sex			−1.41 (303)^a^	.16
Male	181 (59.34)	13.40 (6.07)		
Female	124 (40.66)	14.39 (5.93)		
Age (y)			3.16 (3, 301)^b^	.03^c^
18‐40	31 (10.16)	11.94 (6.93)		
41‐50	66 (21.64)	12.87 (6.82)		
51‐60	120 (39.34)	13.78 (5.90)		
61‐65	88 (28.85)	15.19 (4.89)		
Education level			5.57 (2, 302)^b^	.004^c^
Junior high school or below	223 (73.11)	14.45 (5.62)		
Senior high school or technical secondary school	55 (18.03)	12.55 (7.21)		
College and above	27 (8.85)	11.00 (5.53)		
Occupational status			4.74 (2, 302)^b^	.009^c^
Out of work	88 (28.85)	14.97 (6.46)		
Retirement	151 (49.51)	13.91 (5.56)		
Incumbency	66 (21.64)	12.00 (6.09)		
Marital status			0.77 (2, 302)^b^	.46
Married	258 (84.59)	13.88 (5.99)		.46
Unmarried	18 (5.90)	12.11 (6.01)		
Divorced or widowed	29 (9.51)	14.10 (6.38)		
Religious belief			3.31 (303)^a^	.001^c^
Yes	187 (61.31)	14.74 (5.24)		
No	118 (38.69)	12.31 (6.85)		
Residence			0.04 (2, 302)^b^	.96
With the family	298 (97.70)	13.82 (6.05)		
Live alone	4 (1.31)	13.25 (3.95)		
The rest	3 (0.98)	13.00 (7.00)		
Current address			4.84 (2, 302)^b^	.009^c^
Countryside	137 (44.92)	14.96 (5.54)		
Cities and towns	99 (32.46)	12.70 (6.75)		
Municipalities	69 (22.62)	13.07 (5.47)		
Medical payment methods			3.43 (2, 302)^b^	.05
Medical insurance	273 (89.51)	13.60 (6.06)		
Farmer insurance	31 (10.16)	15.90 (5.12)		
Self-financed	1 (0.33)	4.00 (—^d^)		
Disease-induced burdens			2.46 (2, 302)^b^	.09
Very heavy	85 (27.87)	14.02 (6.05)		
General	211 (69.18)	13.90 (5.96)		
Light	9 (2.95)	9.44 (6.29)		
Self-care ability			8.64 (2, 302)^b^	<.001^c^
Self-care	259 (84.92)	13.35 (5.82)		
Partially self-care	38 (12.46)	15.29 (6.36)		
Unable self-care	8 (2.62)	21.38 (5.07)		
Primary disease			6.06 (3, 301)^b^	.001^c^
DN^e^	59 (19.34)	16.44 (6.23)		
HN^f^	105 (34.43)	13.94 (5.58)		
CG^g^	68 (22.30)	12.35 (5.77)		
The rest	73 (23.93)	12.81 (6.09)		
Dialysis duration (y)			0.97 (2, 302)^b^	.38
<1	55 (18.03)	13.04 (6.27)		
1‐5	127 (41.64)	14.32 (5.62)		
>5	123 (40.33)	13.61 (6.31)		
Dialysis frequency			1.40 (2, 302)^b^	.25
3 times a week	299 (98.03)	13.83 (5.98)		
2 times a week	4 (1.31)	15.25 (8.62)		
The rest	2 (0.66)	7.00 (5.66)		
Type of medication			3.37 (2, 302)^b^	.04^c^
1‐3 species	154 (50.49)	13.88 (6.16)		
4‐7 species	135 (44.26)	13.29 (5.59)		
7 or more	16 (5.25)	17.38 (7.27)		

a *t* test.

b *F* test.

c Indicates significant differences (*P*<.05).

d Not applicable.

e DN: diabetic nephropathy.

f HN: hypertensive nephropathy.

g CG: chronic glomerulonephritis.

**Table 2.** Correlations of variables in patients undergoing hemodialysis (N=305).

Variables	Mean (SD)	1	2	3	4	5	6
1. Demoralization	13.80 (6.02)	1	—^a^	—	—	—	—
2. Trait mindfulness	45.44 (16.42)	−0.78^b^	1	—	—	—	—
3. Confrontation	18.31 (3.42)	−0.64^b^	0.50^b^	1	—	—	—
4. Avoidance	15.96 (3.67)	0.72^b^	−0.68^b^	−0.42^b^	1	—	—
5. Resignation	12.24 (2.93)	0.56^b^	−0.62^b^	−0.42^b^	0.51^b^	1	—
6. Symptom burden	142.19 (54.19)	0.62^b^	−0.62^b^	−0.31^b^	0.57^b^	0.52^b^	1

a Values in the upper triangle are omitted due to symmetry.

b *P*<.001.

#### Multiple Regression Analysis of Demoralization

Multiple regression analysis showed that controlling for sociodemographic variables, trait mindfulness (β=−0.34, *P*<.001), confrontation coping (β=−0.30, *P*<.001), and occupation (β=−0.09, *P*=.01) negatively predicted demoralization scores, whereas the avoidance coping dimension (β=0.27, *P*<.001) and symptom burden (β=0.15, *P*<.001) positively predicted demoralization scores. The above factors predicted 76% of the overall variance in demoralization, *F*_12,292_=57.60, *P*<.001. More details are provided in Section S1-3 in Multimedia Appendix 1.

#### Results of Moderated Mediation Analysis

Based on the regression analysis results, 2 moderated mediation models were constructed separately, each using either the confrontation or the avoidance dimension as the mediator.

##### Confrontation Coping as a Mediator

**Figure 3.**
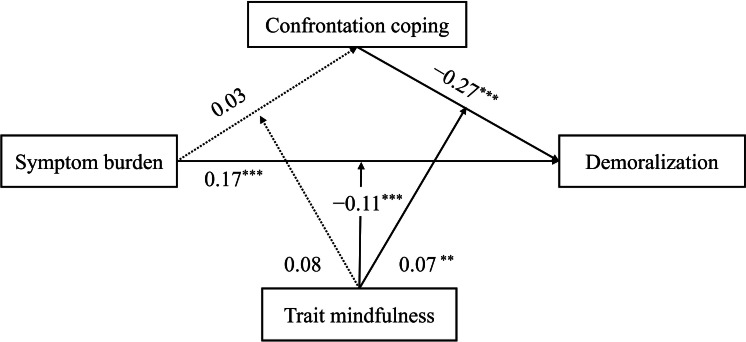
Moderated mediation model with confrontation coping as the mediator. All paths are represented, with solid lines indicating significant associations (*P*<.05) and dotted lines indicating nonsignificant paths. ^**^P<.01, ^***^P<.001*.*

The direct association between symptom burden and demoralization was significant (β=0.17, 95% CI 0.11 to 0.23). Confrontation coping negatively predicted demoralization (β=−0.27, 95% CI −0.33 to −0.21), but symptom burden did not predict confrontation coping (β=0.03, 95% CI −0.09 to 0.15). Trait mindfulness significantly moderated the relationship between symptom burden and demoralization (β=−0.11, 95% CI −0.16 to −0.06). Additionally, trait mindfulness significantly moderated the relationship between confrontation coping and demoralization (β=0.07; 95% CI 0.02 to 0.12; Figure 3, with full statistical details provided in Section S1-4, Table S4 in Multimedia Appendix 1.

##### Avoidance Coping as a Mediator

**Figure 4.**
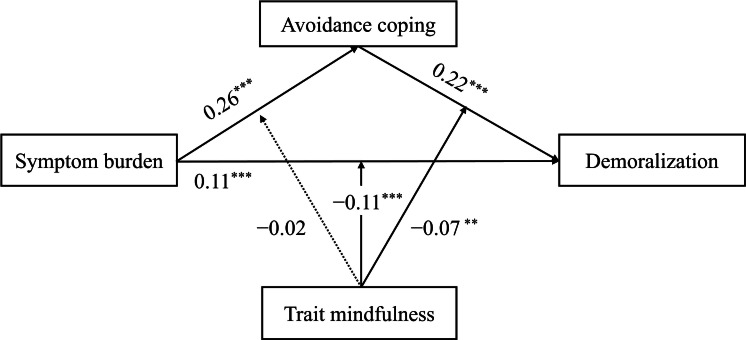
Moderated mediation model with avoidance coping as the mediator. All paths are represented, with solid lines indicating significant associations (*P*<.05) and dotted lines indicating nonsignificant paths. ^**^P<.01*,* ^***^P<.001*.*

The results showed that symptom burden was positively associated with demoralization (β=0.11, 95% CI 0.04 to 0.17) and avoidance coping (β=0.26, 95% CI 0.14 to 0.38), and avoidance coping positively predicted demoralization (β=0.22, 95% CI 0.15 to 0.28). Trait mindfulness significantly moderated the relationship between symptom burden and demoralization (β=−0.11, 95% CI −0.17 to −0.05). Additionally, trait mindfulness significantly moderated the relationship between avoidance coping and demoralization (β=−0.07, 95% CI −0.12 to −0.01; Figure 4, with full statistical details provided in Section S1-4, Table S5 in Multimedia Appendix 1.

### Qualitative Findings

Data analysis yielded 4 key themes: the psychological experience of demoralization, barriers to the alleviation of demoralization, facilitators of demoralization alleviation, and intervention needs. Sixteen subthemes were identified (Sections S1-S5 in Multimedia Appendix 1). Overall, 12 out of 14 (85.7%) interviewees reported symptoms that impacted their psychological and physical health, ranging from itching and fatigue to insomnia and pain. For example, participant N2 stated, “My skin itches a lot, and I always feel very uncomfortable.” Similarly, participant N4 reported, “After dialysis, I personally feel a bit sluggish, I can’t sleep well, my bones feel slightly deformed, and the soles of my feet hurt so much that I can’t walk.”

Furthermore, 6 out of 14 (42.9%) interviewees expressed the need for psychological interventions that are simple, intuitive, and easy to understand. Participants emphasized a preference for interventions that could be learned independently and practiced at home with minimal cognitive or physical burden. As participant N1 explained, “For mindfulness exercises online, it should be simple and easy to do, without requiring too much extra time and energy.” Similarly, participant N10 remarked, “The exercises you suggest shouldn’t be too complicated. I’m getting older, so if there are videos or pictures, it would be easier for me.” Participant N14 further highlighted concerns about accessibility, stating, “I don’t quite understand what you’re saying, but if someone can teach me, I’d be willing to try. After all, I can’t travel far right now, so learning something at home would be good for me. I just worry that the online materials might be too complicated for me to learn. I haven’t read much, so if it’s too complex, I might not be able to grasp it.” Taken together, these findings indicate that patients experiencing substantial symptom burdens not only face ongoing psychological distress but also express a clear demand for accessible, low-threshold, and home-based psychological interventions to help alleviate demoralization.

In addition to the aforementioned factors, the qualitative interviews revealed that stigma, social isolation, and lifestyle changes acted as barriers to the improvement of demoralization, whereas family support, social support, peer support, and confrontation coping served as facilitators.

### Study 2: Effects of MMI

#### Overview

A CONSORT-eHEALTH (Consolidated Standards of Reporting Trials of Electronic and Mobile Health Applications and Online TeleHealth) checklist (Checklist 1) illustrates participant flow, including exclusions, exposure classification, and retention of eligible patients by condition.

Among the 72 randomized participants, 4 participants in the intervention group discontinued the study due to insufficient adherence to the 42-day protocol, yielding a dropout rate of 11.1%. All withdrawals occurred during the second week of the intervention. Qualitative feedback indicated that participants found the daily 10- to 15-minute mindfulness practice and check-in requirement burdensome, particularly on dialysis days or when experiencing fatigue, and that the 2 weekly make-up sessions did not sufficiently accommodate these fluctuations in physical conditions.

Intervention usage was defined as the completion of the daily core practice over the 6-week intervention, with up to 2 make-up sessions permitted per week. Participants in the intervention group completed a mean of 37.3 practice days (6.1, SD 13.19 d/wk), and 32 out of 36 (88.9%) participants met the predefined criterion for full intervention usage.

#### Effectiveness of the MMI

Demographic and clinical characteristics at baseline did not differ significantly between the intervention group and the control group (*P* values>.05; Sections S1-S6, Table S8 in Multimedia Appendix 1).

Significant group × time interaction effects were observed in the following outcome variables: demoralization (*F*_1,70_=93.63, *P*<.001), trait mindfulness (*F*_1,70_=23.38, *P*<.001), confrontation coping (*F*_1,70_=106.28, *P*<.001), avoidance coping (*F*_1,70_=95.33, *P*<.001), total symptom burden (*F*_1,70_=44.55, *P*<.001), and the distress dimension of symptom burden (*F*_1,70_=93.30, *P*<.001).

**Figure 5.**
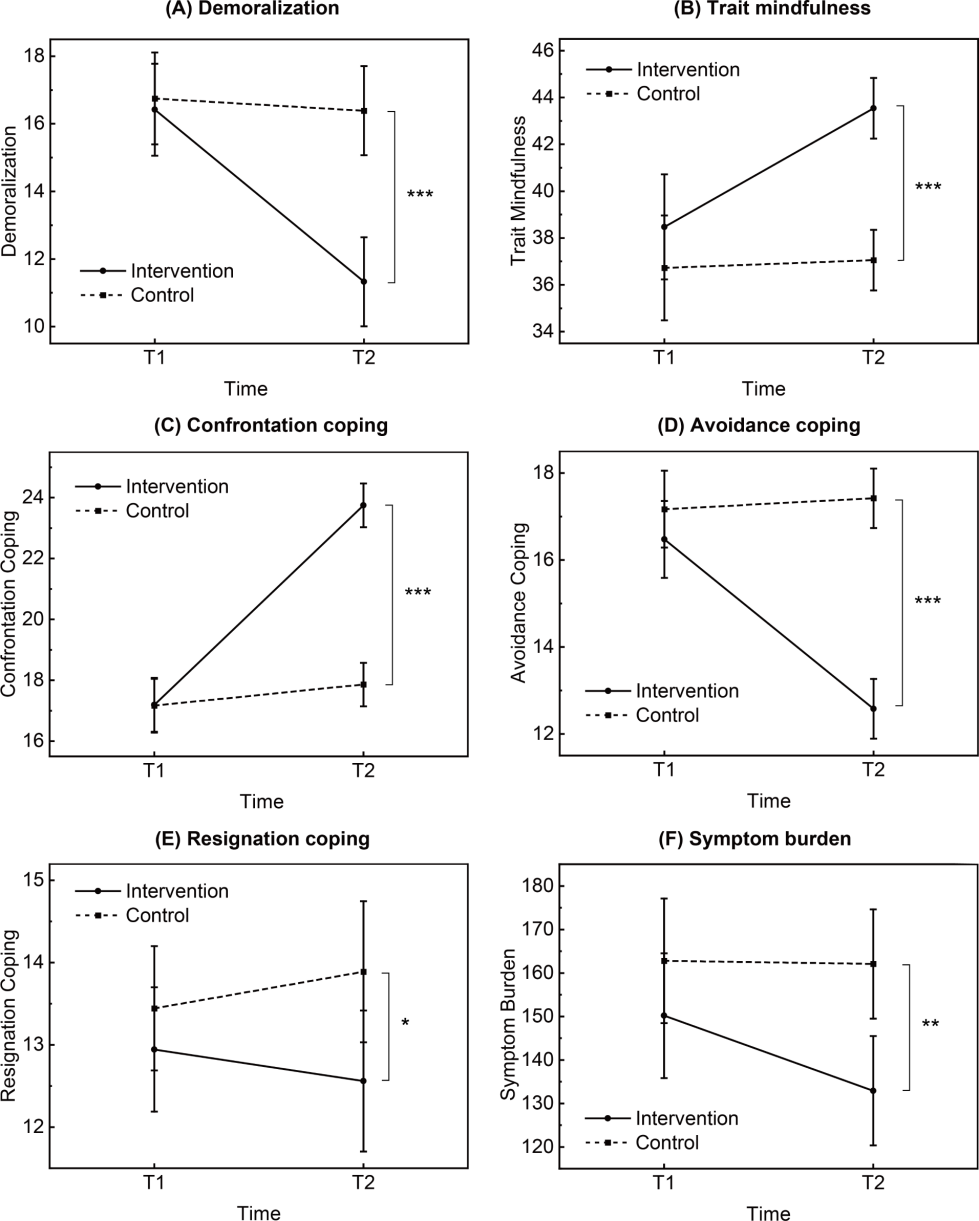
Effects of the mobile-based mindfulness intervention (MMI) in patients receiving hemodialysis. Panels show group × time interactions for (A) demoralization, (B) trait mindfulness, (C) confrontation coping, (D) avoidance coping, (E) resignation coping, and (F) symptom burden. Error lines represent 95% CI. T1: baseline; T2: postintervention. ^*^P<.05*,* ^**^P<.01, ^***^P<.001.

Simple effects analysis indicated that at the postintervention assessment, compared with participants in the control group, those in the intervention group exhibited significantly lower demoralization (mean difference [MD] −5.06, 95% CI −6.92 to −3.20; *P*<.001; Figure 5A), avoidance coping (MD −4.84, 95% CI −5.81 to −3.87; *P*<.001; Figure 5D), resignation coping (MD −1.33, 95% CI −2.54 to −0.12; *P*=.03; Figure 5E), symptom burden (MD −29.15, 95% CI −46.93 to −11.37; *P*=.002; Figure 5F), significantly higher trait mindfulness (MD 6.48, 95% CI 4.65 to 8.31; *P*<.001; Figure 5B), and confrontation coping (MD 5.89, 95% CI 4.88 to 6.90; *P*<.001; Figure 5C). Detailed statistical parameters, including exact test statistics and effect sizes (partial eta-squared), are presented in Section S1-6, Table S9 in Multimedia Appendix 1.

#### Sensitivity Analyses

To evaluate the robustness of the primary findings, a sensitivity analysis was performed on the 68 participants who completed the full trial (n=32 in the intervention group, n=36 in the control group). The results were highly consistent with the primary intention-to-treat analyses: at postintervention, the intervention group showed significant improvements across all outcomes, including decreased demoralization, symptom burden, and maladaptive coping strategies (avoidance and resignation), as well as increased trait mindfulness and adaptive coping (confrontation) compared to the control group. Detailed statistical parameters for this sensitivity analysis are presented in Section S1-6, Table S10 in Multimedia Appendix 1.

## Discussion

### Principal Findings

This study provides converging evidence from quantitative analyses, qualitative exploration, and an RCT regarding the mechanism underlying demoralization and the effectiveness of a tailored MMI among patients receiving hemodialysis. By integrating convergent results, this study extends the current evidence in 3 important ways. First, it identifies a potential psychological pathway linking symptom burden to demoralization, with trait mindfulness functioning as a potential buffering role. Second, it provides preliminary evidence supporting the effectiveness of a 6-week MMI in reducing demoralization and improving psychological outcomes in an understudied clinical population. Third, this study demonstrates the value of developing patient-tailored digital psychological interventions, showing that mindfulness-based approaches can be adapted to the specific clinical context of hemodialysis.

The findings from study 1 provide important insights into the psychological processes underlying demoralization among patients receiving hemodialysis. Symptom burden was positively associated with demoralization, and this association was partly explained by avoidance coping. These findings are consistent with previous studies [18,19] and the stress system model [34], which suggest that patients’ responses to persistent symptoms may represent a critical psychological pathway linking physical burden to existential distress. For patients receiving hemodialysis, recurrent symptoms, treatment dependency, and uncertainty regarding disease progression may increase psychological distress and reinforce avoidance coping, such as disengagement from illness-related challenges.

Trait mindfulness emerged as a protective factor that attenuated the associations between symptom burden and avoidance coping with demoralization. This finding is consistent with previous research suggesting that mindfulness may enhance psychological flexibility by promoting nonjudgmental awareness of internal experiences and reducing automatic emotional reactions [43,44]. By promoting acceptance and self-distancing, mindfulness may reduce avoidance-based regulation and negative affect [45], thereby buffering the adverse effects of symptom burden and maladaptive coping on demoralization. However, the absence of moderation on the symptom burden-avoidance pathway suggests that mindfulness may be more effective in modifying emotional responses following avoidance coping rather than preventing the initial emergence of avoidance tendencies under chronic stress [46,47]. These mechanism-oriented findings provided the theoretical foundation for the subsequent MMI intervention, highlighting the importance of cultivating mindfulness skills in patients receiving hemodialysis.

Building on the findings of study 1, the RCT in study 2 showed that the MMI was associated with greater reductions in demoralization and symptom burden, as well as greater increases in trait mindfulness and adaptive coping, compared with usual care. These findings are consistent with growing evidence that online MBIs can improve psychological well-being and alleviate emotional distress among people with chronic illness [48]. Usual care, which primarily focuses on biomedical management, may be insufficient to address the psychological consequences of persistent symptoms and long-term treatment dependence [49]. This is particularly important because demoralization involves the loss of meaning, hope, and perceived competence rather than emotional distress alone.

The intervention effects are broadly consistent with the psychological pathways identified in study 1. The observed increase in trait mindfulness and improvement in coping patterns in the MMI group may therefore represent plausible pathways through which the intervention reduced demoralization. Mindfulness practices may help patients notice physical sensations and illness-related thoughts with less judgment and reactivity, thereby reducing reliance on avoidance-based coping. The intervention also incorporated self-compassion-oriented practices, including body scanning and mindful breathing. As self-criticism and hopelessness are closely related to demoralization, self-compassion may have complemented the mindfulness and coping-related processes described above [50,51]. For patients receiving hemodialysis, who may experience bodily loss of control and treatment-related dependence, these practices may facilitate a more accepting and less self-critical relationship with their bodies.

Beyond intervention efficacy, this study supports the feasibility of delivering mindfulness-based psychological support through a mobile platform for patients receiving hemodialysis. Although traditional mindfulness programs can be effective [28], their implementation may be challenging because of dialysis schedules, physical fatigue, transportation burden, and limited access to psychological services [29]. In contrast, the mobile-based format allowed patients to complete brief daily practices at their convenience, thereby reducing these logistical barriers. Informed by qualitative findings, the intervention used brief videos and easy-to-understand text-image materials to meet patients’ preferences for simple, low-threshold, and accessible psychological support [52]. In addition, the WeChat-based peer support component may have further enhanced engagement and psychological benefits. Previous research has suggested that peer and social support can improve psychological adjustment among individuals with chronic illnesses [53,54] by reducing isolation [55] and strengthening perceived support [56]. Thus, combining structured mindfulness practice with patient-centered digital delivery and peer interaction may provide a practical approach to integrating psychosocial care into routine dialysis management.

Despite these strengths, the study has some limitations. First, the absence of an active or attention-matched control group means that the specific efficacy of the mindfulness components cannot be fully distinguished from nonspecific effects, such as general clinical attention and expectancy. Future studies should use active control conditions or dismantling designs to isolate the unique effects of the mindfulness modules. Second, while peer interaction via the WeChat group was designed to enhance engagement [57], it introduced a potential confounding cointervention. Because the intensity and quality of this peer interaction were not systematically measured, we could not separate the psychological benefits of social/peer support from those of pure mindfulness practice. Future studies should incorporate structured peer-support protocols and objective interaction indicators to clarify the relative contribution of the peer component. Third, the short follow-up period and reliance on self-reported outcomes limited the assessment of the durability and broader clinical impact of the intervention [58]. Future studies should include longer follow-up assessments [59,60] and incorporate clinician-rated, behavioral, or other objective measures to evaluate the maintenance and robustness of intervention effects. Fourth, adherence was assessed using check-in completion because the platform could not capture objective engagement indicators; however, this may not fully reflect actual intervention use. Moreover, early attrition occurred during the second week, mainly due to the burden of daily practice during dialysis days or periods of fatigue. Future digital interventions should incorporate automatic behavioral tracking and adaptive adherence strategies to better capture engagement and accommodate patients’ fluctuating physical conditions.

### Conclusions

This study provides evidence that a theory-informed, hemodialysis-specific, MMI can effectively reduce demoralization and improve psychological outcomes among patients receiving long-term dialysis. By integrating converging evidence, this study extends current knowledge on digital psychological interventions by highlighting the potential of patient-tailored mindfulness approaches to address the psychological consequences of chronic disease beyond routine biomedical management. The findings highlight the potential of scalable mobile-based interventions to support the integration of psychosocial care into nephrology practice, particularly for populations facing barriers to accessing traditional psychological services. Future research should examine the long-term effectiveness, implementation strategies, and cost-effectiveness of integrating digital psychological care into routine chronic disease management.

## Supplementary material

10.2196/90324 Multimedia Appendix 1 Interview outline and supplement to data analysis details.

10.2196/90324 Checklist 1 CONSORT-eHEALTH checklist (V 1.6.1).

## Abbreviations

CONSORT-eHEALTH: Consolidated Standards of Reporting Trials of Electronic and Mobile Health Applications and Online TeleHealth
DS-II: Demoralization Scale-II
MBI: mindfulness-based intervention
MD: mean difference
MMI: mobile-based mindfulness intervention
RCT: randomized controlled trial

## References

[1] Coluzzi F (2018). Assessing and treating chronic pain in patients with end-stage renal disease. Drugs.

[2] Samoudi AF, Marzouq MK, Samara AM, Zyoud SH, Al-Jabi SW (2021). The impact of pain on the quality of life of patients with end-stage renal disease undergoing hemodialysis: a multicenter cross-sectional study from Palestine. Health Qual Life Outcomes.

[3] Wu YH, Hsu YJ, Tzeng WC (2022). Correlation between physical activity and psychological distress in patients receiving hemodialysis with comorbidities: a cross-sectional study. Int J Environ Res Public Health.

[4] Kissane DW (2001). Demoralisation: its impact on informed consent and medical care. Med J Aust.

[5] Zhang H, Yang L, Xing X, Huang Y, Lv X (2025). Incidence and influencing factors of demoralization syndrome in elderly maintenance hemodialysis patients: a multi-center cross-sectional study. Int J Gen Med.

[6] Moreira MB, Cavalli NP, Righi NC, Schuch FB, Signori LU, da Silva AMV (2023). Quality of life and functional capacity in depressive patients on hemodialysis: a systematic review and meta-analysis. Braz J Med Biol Res.

[7] Sawicki GS, Sellers DE, Robinson WM (2008). Self-reported physical and psychological symptom burden in adults with cystic fibrosis. J Pain Symptom Manage.

[8] Gerstorf D, Schilling OK, Pauly T (2023). Long-term aging trajectories of the accumulation of disease burden as predictors of daily affect dynamics and stressor reactivity. Psychol Aging.

[9] An E, Lo C, Hales S, Zimmermann C, Rodin G (2018). Demoralization and death anxiety in advanced cancer. Psychooncology.

[10] Philipp R, Mehnert A, Müller V, Reck M, Vehling S (2020). Perceived relatedness, death acceptance, and demoralization in patients with cancer. Support Care Cancer.

[11] Folkman S, Lazarus RS, Dunkel-Schetter C, DeLongis A, Gruen RJ (1986). Dynamics of a stressful encounter: cognitive appraisal, coping, and encounter outcomes. J Pers Soc Psychol.

[12] Exner A, Kampa M, Finke JB (2023). Repressive and vigilant coping styles in stress and relaxation: evidence for physiological and subjective differences at baseline, but not for differential stress or relaxation responses. Front Psychol.

[13] Ma F, Zheng W, Wang W, Zhang N, Liu Y (2024). The mediating role of medical coping modes between stigma and quality of life in patients undergoing lung cancer surgery: a cross-sectional study. BMC Cancer.

[14] Huda N, Shaw MK, Chang HJ, Putri ST, Pranata S (2024). The mediating role of coping styles in the relationship between fear of COVID-19 and mental health problems: a cross-sectional study among nurses. BMC Public Health.

[15] Pelekanakis A, Doré I, Sylvestre MP, Sabiston CM, O’Loughlin J (2022). Mediation by coping style in the association between stressful life events and depressive symptoms in young adults. Soc Psychiatry Psychiatr Epidemiol.

[16] Shiraly R, Roshanfekr A, Asadollahi A, Griffiths MD (2024). Psychological distress, social media use, and academic performance of medical students: the mediating role of coping style. BMC Med Educ.

[17] Xiao T, Han S, Li Q (2025). The mediating effect of positive coping in the association between inner strength and demoralization in lung cancer chemotherapy patients. Support Care Cancer.

[18] Hao R, Zhang M, Zuo J, Qi Y, Hu J (2023). Contribution of coping style to the association between illness uncertainty and demoralisation in patients with breast cancer: a cross-sectional mediation analysis. BMJ Open.

[19] Zhang C, Wang F, Kang Z (2025). Effect of symptom burden on demoralization in Chinese lung cancer patients: the mediating roles of family function, resilience, and coping behaviors. Psychooncology.

[20] Carpenter JK, Conroy K, Gomez AF, Curren LC, Hofmann SG (2019). The relationship between trait mindfulness and affective symptoms: a meta-analysis of the Five Facet Mindfulness Questionnaire (FFMQ). Clin Psychol Rev.

[21] Brown KW, Ryan RM (2003). The benefits of being present: mindfulness and its role in psychological well-being. J Pers Soc Psychol.

[22] Sala M, Rochefort C, Lui PP, Baldwin AS (2020). Trait mindfulness and health behaviours: a meta-analysis. Health Psychol Rev.

[23] Chen J, Fu G, Zhou Y, Xu W (2023). Emotion regulation strategies and PTSS among children who experienced an explosion accident: the moderating role of trait mindfulness. Psychol Health Med.

[24] Zhong F, Gu C (2024). The impact of health information echo chambers on older adults avoidance behavior: the mediating role of information fatigue and the moderating role of trait mindfulness. Front Psychol.

[25] Kabat-Zinn J (2003). Mindfulness-based interventions in context: past, present, and future. Clin Psychol Sci Pract.

[26] Al-Ghabeesh SH, Rayan A, Hattab F, Jarrar Y (2022). Mindfulness and psychological distress among hemodialysis patients. Psychol Health Med.

[27] Majeed MH, Ali AA, Sudak DM (2018). Mindfulness-based interventions for chronic pain: evidence and applications. Asian J Psychiatr.

[28] Goyal M, Singh S, Sibinga EMS (2014). Meditation programs for psychological stress and well-being: a systematic review and meta-analysis. JAMA Intern Med.

[29] Jesus S, Antunes JP, Andretta I, Russel T, Mello L, Chiodelli R (2025). Comparison of face-to-face and synchronous online mindfulness-based interventions: a quasi-experimental study. Health Psychol Rep.

[30] Thomas Z, Novak M, Platas SGT (2017). Brief mindfulness meditation for depression and anxiety symptoms in patients undergoing hemodialysis: a pilot feasibility study. Clin J Am Soc Nephrol.

[31] Zhu T, Zhang L, Weng W (2025). Effectiveness of an Internet-Based, Self-Guided, Short-Term Mindfulness Training (ISSMT) program for relieving depressive symptoms in the adult population in China: single-blind, randomized controlled trial. J Med Internet Res.

[32] Li Y, Zhang Y, Wang C (2025). Supported mindfulness-based self-help intervention as an adjunctive treatment for rapid symptom change in emotional disorders: a practice-oriented multicenter randomized controlled trial. Psychother Psychosom.

[33] Wang Z, Shalihaer K, Hofmann SG, Feng S, Liu X (2024). The role of attentional control in mindfulness intervention for emotional distress: a randomized controlled trial with longitudinal mediation analyses. Clin Psychol Psychother.

[34] Jiang QJ (2024). Psychological Stress (Stress) System Model [Book in Chinese].

[35] Shapiro SL, Carlson LE, Astin JA, Freedman B (2006). Mechanisms of mindfulness. J Clin Psychol.

[36] Robinson S, Kissane DW, Brooker J (2016). Refinement and revalidation of the Demoralization Scale: the DS-II-external validity. Cancer.

[37] Shen XH, Jiang QJ (2000). Report on application of Chinese version of MCMQ in 701 patients. Chin J Behav Med Sci.

[38] Danquah FVN, Zimmerman L, Diamond PM, Meininger J, Bergstrom N (2010). Frequency, severity, and distress of dialysis-related symptoms reported by patients on hemodialysis. Nephrol Nurs J.

[39] Preacher KJ, Hayes AF (2008). Asymptotic and resampling strategies for assessing and comparing indirect effects in multiple mediator models. Behav Res Methods.

[40] Aiken LS, West SG (1991). Multiple Regression: Testing and Interpreting Interactions.

[41] Hsieh HF, Shannon SE (2005). Three approaches to qualitative content analysis. Qual Health Res.

[42] Urbaniak GC, Plous PS Research Randomizer.

[43] Garland EL, Farb NA, Goldin P, Fredrickson BL (2015). Mindfulness broadens awareness and builds eudaimonic meaning: a process model of mindful positive emotion regulation. Psychol Inq.

[44] Raugh IM, Berglund AM, Strauss GP (2024). Implementation of mindfulness-based emotion regulation strategies: a systematic review and meta-analysis. Affect Sci.

[45] Petrova K, Nevarez MD, Waldinger RJ, Preacher KJ, Schulz MS (2021). Self-distancing and avoidance mediate the links between trait mindfulness and responses to emotional challenges. Mindfulness (N Y).

[46] Eustis EH, Hayes-Skelton SA, Roemer L, Orsillo SM (2016). Reductions in experiential avoidance as a mediator of change in symptom outcome and quality of life in acceptance-based behavior therapy and applied relaxation for generalized anxiety disorder. Behav Res Ther.

[47] Treanor M, Barry TJ (2017). Treatment of avoidance behavior as an adjunct to exposure therapy: insights from modern learning theory. Behav Res Ther.

[48] Johnson E, Corrick S, Isley S (2024). Mind-body internet and mobile-based interventions for depression and anxiety in adults with chronic physical conditions: a systematic review of RCTs. PLOS Digit Health.

[49] Fava GA, Patierno C, Sonino N, Cosci F (2024). Key psychosocial issues in medical care. Acta Psychiatr Scand.

[50] Goldberg SB, Davidson RJ (2024). Contemplative science comes of age: looking backward and forward 20 years after Baer (2003). Clin Psychol (New York).

[51] Inwood E, Ferrari M (2018). Mechanisms of change in the relationship between self-compassion, emotion regulation, and mental health: a systematic review. Appl Psychol Health Well Being.

[52] Pichayapinyo P, Sompopcharoen M, Thiangtham W (2024). Perceptions of the 2D short animated videos for literacy against chronic diseases among adults with diabetes and/or hypertension: a qualitative study in primary care clinics. BMC Prim Care.

[53] Dave S, Kim SC, Beaver S (2024). Peer support in adolescents and young adults with chronic or rare conditions in northern America and Europe: targeted literature review. J Pediatr Nurs.

[54] Thompson DM, Booth L, Moore D, Mathers J (2022). Peer support for people with chronic conditions: a systematic review of reviews. BMC Health Serv Res.

[55] Novick TK, Osuna M, Emery C (2024). Patients’ perspectives on health-related social needs and recommendations for interventions: a qualitative study. Am J Kidney Dis.

[56] Washington TR, Hamler TC (2025). Recommendations to advance social connectedness in end stage kidney disease care in a post-COVID-19 era. Am J Kidney Dis.

[57] Eliacin J, Patterson SM, Mendez DM (2023). Findings from a peer-facilitated, social isolation intervention in the Veterans Health Administration healthcare system: a mixed-methods, pilot feasibility study. J Gen Intern Med.

[58] Salzler M, Saithna A, Cote MP, Matzkin E, Rossi MJ (2025). Lost but not forgotten: how to manage follow-up loss in clinical research. Arthroscopy.

[59] Yavuz Sercekman M (2024). Exploring the sustained impact of the Mindfulness-Based Stress Reduction program: a thematic analysis. Front Psychol.

[60] Kuyken W, Hayes R, Barrett B (2015). Effectiveness and cost-effectiveness of mindfulness-based cognitive therapy compared with maintenance antidepressant treatment in the prevention of depressive relapse or recurrence (PREVENT): a randomised controlled trial. Lancet.

