# Supplementary material for: A Mobile-Based Mindfulness Intervention for Reducing Demoralization in Patients on Maintenance Hemodialysis: Randomized Controlled Trial
Source: J Med Internet Res. 2026 Aug 7;28:e90324. doi: 10.2196/90324 (PMC13451012; doi:10.2196/90324)
Supplement: Supplementary file 2 [file jmir-v28-e90324-s001.doc]

**A Mobile-Based Mindfulness Intervention for Reducing Demoralization in Patients on Maintenance Hemodialysis: Randomized Controlled Trial**

**Multimedia Appendix 1**

Contents

Multimedia Appendix 1-1	2

Multimedia Appendix 1-2	3

Multimedia Appendix 1-3	6

Multimedia Appendix 1-4	8

Multimedia Appendix 1-5	11

Multimedia Appendix 1-6	19

**Multimedia Appendix 1-1**

**The interview outline**

They were asked about:

(a) How did you feel when it was confirmed that you would be on hemodialysis? How did you feel when you decided to go on hemodialysis?
(b) Have you ever had a positive experience in your daily life? If so, please describe. In what situations are you able to experience positive thoughts? How does this affect you? Do you think positive thinking can help you to alleviate these feelings?
(c) What stresses are you experiencing at this stage? What would you like help with? Do you know anything about positive thinking on the Internet? Are you willing to try it?

**Multimedia Appendix 1-2**

The research team consisted of one psychology professor, one associate professor of nursing, one psychological counselor with more than 15 years of experience in mindfulness implementation, one hemodialysis physician, two specialized hemodialysis nurses, and one graduate student in psychology. Five experts from relevant fields were invited to consult, discussing and reaching consensus on the intervention’s effectiveness and scientific validity. The selection criteria for the experts were: (1) research or work experience in mindfulness, hemodialysis nursing, or psychology; (2) bachelor’s degree or higher, with a mid-level or higher professional title; and (3) voluntary participation. The purpose of the expert consultation and a draft of the intervention were sent to the experts via email. The average judgment value from the five experts was 0.92, the familiarity value was 0.8, and the expert authority value was 0.86, indicating a high level of acceptability. The experts’ feedback and suggestions were integrated into a revised version of the intervention (Table S1).

***Table S1.*** Revised Positive Thinking Intervention Program for MHD Patients

| weekly | Theme of intervention | concrete content |
| --- | --- | --- |
| First week | Positive Thinking Foundation and Intention Setting | 1. **Knowledge block**: Through a short video form, introduce the basic principles of positive thinking and IAA model (Positive Thinking Ability Model), explain the importance of intention, attention and attitude to positive thinking practice.  2. **Practice segment**: Through vision meditation on day 1, patients are guided to identify and express their initial intentions and goals for engaging in positive thinking practice. Days 2-7 will be devoted to the practice of eating raisins with positive thinking.  3. **Punch Card Board**: Share your goals for positive thinking practice and your intention to participate in the dialysis positive thinking journey. |
| Second week | Body perception and attitude development | 1. **Knowledge block**: Through the form of short videos, learn the purpose of body scanning and the practice of Tzu Chi Chi Zen, the main points of practice, and the precautions of practice.  2.**Practice segment**: Body Scanning + Compassionate Zen, through the body scanning exercise, MHD patients are guided to observe all parts of the body in detail, paying special attention to the kidneys, arm fistula, and other parts of the body related to dialysis, and the exercise guide adds self-care components to increase the patient's sensitivity and acceptance of the body's symptoms. The exercise guided patients to observe their own experiences, especially discomfort and symptoms, in a non-judgmental, accepting and compassionate manner.  3. **Punch card board**: share your feelings during body scanning practice and compassion meditation practice. |
| Third week | Attention to the Acceptance Cultivation of Swimming | 1.**Knowledge block**: Through short videos, learn about mindfulness of breathing and three-step breathing space. Learn the purpose, points, and precautions of the practice of Positive Breathing and Three-Step Breathing Space.  2.**Practice segment**: Body Scanning/Positive Breathing + Three-Step Breathing Space guides the patient to cultivate an attitude of acceptance of the moment when attention wanders through the practice of Positive Breathing. By being aware of the present moment of attention wandering, the patient accepts any experience that arises in the present moment. Through the practice, the patient's cognitive flexibility is improved, the patient's tolerance of symptom burden is increased, and the patient's avoidance behaviors are reduced.  3.**Punch Card Board**: Share your feelings during the Body Scanning / Positive Breathing + Three Step Breathing Space practice. |
| Fourth week | Positive Thinking Response: treating thoughts as thoughts | 1. **Knowledge board**: through the short video form, learn the purpose, type, practice points, practice notes of positive thinking walking practice. Learning the content of treating thoughts as thoughts and how to do so in dialysis life.  2. **Exercise segment**: Positive Breathing / Body Scanning / Positive Walking Triple Choice + 3-step Breathing Space, in the practice of Positive Thinking Walking, becoming aware of the emergence of one's own thoughts, and being able to recognize that thoughts are just a product of the inner self. Explore the application of positive thinking exercises in dealing with dialysis-related challenges, and practice how to apply positive thinking coping strategies in the face of symptom burden and psychological stress through specific cases and role-plays.  3. **Punch Card Board**: Share your feelings during the Positive Thinking Breathing / Body Scanning / Positive Thinking Walking Triple Choice + 3-Step Breathing Space Exercise. |
| Fifth week | Trait Positive Thinking Cultivation and Practice | 1. **Knowledge Board**: Learn about awareness and acceptance and the nine attitudes of positive thinking in the form of short videos.  2. **Practice Board**: Positive Breathing/Body Scanning/Positive Walking (choose one out of three) + Compassionate Zen exercises, discussing how to apply intention, attention, and attitudes to these exercises in order to enhance awareness and acceptance of experience. Provide in-depth positive thinking exercises to help patients enhance trait positive thinking in their daily lives.  3. **Punch Card Board**: Share your feelings during Positive Breathing/Body Scanning/Walking with Positive Thoughts (choose one of the three) + Tzu Chi Chi Zen practice. 4. |
| Sixth week | Taking stock and looking ahead | 1. **Knowledge Board**: Learn how to integrate positive thinking into dialysis life through a short video format. Summarize the key learning points and personal growth experiences in the process of positive thinking intervention, and emphasize the role of intention, attention and attitude in this process.  2. **Practice segment**: Integrating Positive Thinking into Dialysis Life, Positive Thinking Breathing Exercises during Dialysis, Body Scanning Exercises, Positive Thinking Eating, Positive Thinking Conversations, and Dialysis Journal. Discuss how to consistently integrate mindfulness practices into future life, including when facing the challenges of chronic illnesses such as dialysis.  3. **Punch card board**: share feelings about integrating positive thinking into future dialysis life. |

***Note.*** The intervention takes the form of completing tasks through the WeChat mini-program punch card software, 15-25 minutes per day, and WeChat group communication and Q&A.

**Multimedia Appendix 1-3**

To investigate the factors affecting demoralization in MHD patients, the dependent variable was the score of demoralization, and the eight factors that were significant in the univariate analysis (age, education, occupational status, religion, place of residence, self-care, primary disease, and types of medication), as well as the trait mindfulness, the confronting coping dimension, the avoiding coping dimension, the yielding coping dimension, and the symptom burden were used as the independent variables in a multiple regression analysis. The values of the dummy variables are shown in Tables S2.

**Table S2.** *Division table*

| Variable | variable name | | | form of assignment |
| --- | --- | --- | --- | --- |
| Age | X_1_ |  | | 18-40year=1; 41-50yera=2;  51-60year=3; 61-65year=4 |
| Education level | X_2_ | |  | Junior high school or below=1; Senior high school or technical secondary school=2；College and above=3 |
| Occupational status | X_3_ | | out of work(X_31_)  retirement (X_32_)  incumbency (X_33_) | X_32_=0; X_33_=0;(consultation)  X_32_=1; X_33_=0;  X_32_=0; X_33_=1; |
| Religious belief | X_4_ | |  | Yes=1; No=2 |
| Current address | X_5_ | | Countryside (X_51_)  Cities and towns (X_52_)  Municipalities (X_53_) | X_52_=0; X_53_=0; (consultation)  X_52_=1; X_53_=0;  X_52_=0; X_53_=1; |
| Self-care ability | X_6_ | |  | Self-care =1; Partially self-care =2; Unable self-care =3 |
| Primary disease | X_7_ | | DN(X_71_)  HN(X_72_)  CG(X_73_)  the rest（X_74_） | X_72_=0; X_73_=0; X_74_=0;(consultation)  X_72_=1; X_73_=0; X_74_=0;  X_72_=0; X_73_=1; X_74_=0;  X_72_=0; X_73_=0; X_74_=1; |
| Type of medication | X_8_ | |  | 1-3species =1; 4-7species =2;  7 or more =3 |

*Note.* DN=Diabetic nephropathy; HN=hypertensive nephropathy; CG=chronic glomerulonephritis;

The results showed that among MHD patients, trait mindfulness (*β* = -0.34, *p* < 0.001), confrontation coping dimension (*β* = -0.30, *p* <0.001), and occupation (*β* = -0.09, *p* = 0.014) negatively predicted disorientation syndrome scores, whereas avoidance coping dimension (*β* = 0.27, *p* < 0.001), and symptom burden (*β* = 0.15, *p* < 0.001) positively predicted the disorientation syndrome score. The above factors predicted 76% of the overall variance in disorientation syndrome, *F* = 57.60, *p* < 0.001. The diagnosis of covariance showed that the tolerance values of the variables ranged from 0.35 to 0.87, and the VIF values ranged from 1.16 to 2.86, with a small probable line of multicollinearity. The Durbin-Watson value was 1.94, indicating good sample independence. See Table S3 for details.

**Table S3.** *Multivariate regression analysis*

| variable |  | standard error | *t* | *p* | covariance statistics | |
| --- | --- | --- | --- | --- | --- | --- |
|  | *β* |  |  |  | tolerances | VIF |
| (Constant) |  | 0.17 | 7.54 | **<0.001** |  |  |
| Age | -0.00 | 0.01 | -0.03 | 0.979 | 0.63 | 1.59 |
| Education level | 0.06 | 0.02 | 1.71 | 0.088 | 0.64 | 1.57 |
| retirement | -0.06 | 0.03 | -1.64 | 0.101 | 0.58 | 1.73 |
| incumbency | -0.09 | 0.03 | -2.48 | **0.014** | 0.63 | 1.59 |
| religious belief | -0.06 | 0.03 | -1.82 | 0.070 | 0.69 | 1.45 |
| cities and towns | 0.03 | 0.03 | 0.89 | 0.376 | 0.69 | 1.46 |
| municipalities | -0.01 | 0.03 | -0.29 | 0.773 | 0.71 | 1.42 |
| Self-care ability | 0.04 | 0.03 | 1.38 | 0.170 | 0.87 | 1.16 |
| HN | -0.05 | 0.03 | -1.24 | 0.218 | 0.52 | 1.91 |
| CG | -0.08 | 0.04 | -1.94 | 0.054 | 0.52 | 1.90 |
| the rest | -0.02 | 0.04 | -0.57 | 0.566 | 0.51 | 1.97 |
| Type of medication | 0.04 | 0.02 | 1.20 | 0.232 | 0.86 | 1.16 |
| Trait mindfulness | -0.34 | 0.02 | -7.22 | **<0.001** | 0.35 | 2.86 |
| Confrontation coping | -0.30 | 0.03 | -8.33 | **<0.001** | 0.62 | 1.62 |
| Avoidance coping | 0.27 | 0.03 | 6.36 | **<0.001** | 0.45 | 2.25 |
| Resignation coping | -0.01 | 0.02 | -0.15 | 0.877 | 0.55 | 1.83 |
| Symptom burden | 0.15 | 0.03 | 3.91 | **<0.001** | 0.53 | 1.90 |

*Note. R*^2^=0.77;Adjusted *R*^2^=0.76;*F*=57.60; *p*<0.001；*β* is standardized; HN=hypertensive nephropathy; CG=chronic glomerulonephritis;

**Multimedia Appendix 1-4**

**Table S4.** Regression results for the moderated mediation model with confrontation coping as the mediator.

| Predictor variable | *β* | *SE* | *t* | 95%CI |
| --- | --- | --- | --- | --- |
| **Equation 1 Dependent variable: confrontation coping** | | | | |
| Symptom burden (X) | 0.03 | 0.06 | 0.53 | [-0.09, 0.15] |
| Trait mindfulness(U) | 0.25 | 0.03 | 8.26^***^ | [0.19, 0.31] |
| X × U | 0.08 | 0.05 | 1.72 | [-0.01, 0.17] |
| R^2^ |  |  | 0.33 |  |
| *F* |  |  | 12.90^***^ |  |
| **Equation 2 Dependent variable: demoralization** | | | | |
| Symptom burden(X） | 0.17 | 0.03 | 5.47^***^ | [0.11, 0.23] |
| Confrontation coping（M） | -0.27 | 0.03 | -8.86^***^ | [-0.33, -0.21] |
| Trait mindfulness（U） | -0.22 | 0.02 | -11.90^***^ | [-0.25, -0.18] |
| X × U | -0.11 | 0.03 | -4.29^***^ | [-0.16, -0.06] |
| M × U | 0.07 | 0.03 | 2.71^**^ | [0.02, 0.12] |
| R^2^ |  |  | 0.76 |  |
| *F* |  |  | 71.88^***^ |  |

*Note*. The model was controlled for sociodemographic variables. ^**^*p* < 0.01, ^***^*p* < 0.001. SE, standard error; CI, confidence interval.

***Table S5.*** *Regression results for the moderated mediation model with avoidance coping as the mediator.*

| Predictor variable | *β* | *SE* | t | *95%CI* |
| --- | --- | --- | --- | --- |
| **Equation 1 Dependent variable:** **Avoidance coping** | | | | |
| Symptom burden(X) | 0.26 | 0.06 | 4.28^***^ | [0.14, 0.38] |
| Trait mindfulness(U) | -0.24 | 0.03 | -7.78^***^ | [-0.31, -0.18] |
| X × U | -0.02 | 0.05 | -0.32 | [-0.11, 0.08] |
| R^2^ |  |  | 0.53 |  |
| *F* |  |  | 29.89^***^ |  |
| **Equation 2 Dependent variable: demoralization** | | | | |
| Symptom burden(X) | 0.11 | 0.03 | 3.23^**^ | [0.04, 0.17] |
| Avoidance coping(M) | 0.22 | 0.03 | 6.82^***^ | [0.15, 0.28] |
| Trait mindfulness(U) | -0.24 | 0.02 | -12.03^***^ | [-0.28, -0.20] |
| X × U | -0.11 | 0.03 | -3.61^***^ | [-0.17, -0.05] |
| M × U | -0.07 | 0.03 | -2.33^**^ | [-0.12, -0.01] |
| R^2^ |  |  | 0.74 |  |
| *F* |  |  | 64.38^***^ |  |

*Note*. The model was controlled for sociodemographic variables. ^*^*p* < 0.05, ^**^*p* < 0.01, ^***^*p* < 0.001. SE, standard error; CI, confidence interval.

***Simple slope analyses***

According to the simple slope method proposed by Aiken and West, trait positivity was differentiated into high value group (mean + standard deviation), medium value group (mean), and low value group (mean - standard deviation) to analyze the relationship between symptom burden, avoidant coping, and demoralization at different levels of trait mindfulness, as shown in Table S6.

**Table S6.** *Path coefficients of the “symptom burden-* *demoralization” relationship at different levels of trait mindfulness*

| Path | *β* | *SE* | 95%CI |
| --- | --- | --- | --- |
| **Symptom burden-demoralization** | | | |
| Low-TM | 0.23 | 0.05 | ［0.14,0.31］ |
| Middle-TM | 0.11 | 0.03 | ［0.04,0.17］ |
| High-TM | -0.01 | 0.05 | ［-0.10,0.09］ |
| **Avoidance coping** -**demoralization** | | | |
| Low-TM | 0.29 | 0.04 | ［0.21,0.37］ |
| Middle-TM | 0.22 | 0.03 | ［0.15,0.28］ |
| High-TM | 0.14 | 0.05 | ［0.05,0.24］ |

*Note*. TM=Trait mindfulness;

With avoidance coping as the mediator variable, the model controlled for age, education, occupational status, religious credentials, place of residence, self-care status, primary disease, and type of medication used in patients with MHD; *SE* is the standard error; and 95% CI denotes the upper and lower bounds of the 95% confidence interval.

As shown in Figure S1, at the low levels of trait mindfulness, demoralization increased significantly with greater symptom burden (*β* = 0.23, *t* = 4.99, *p* < 0.001). In contrast, at high levels of trait mindfulness, demoralization remained stable and was not significantly associated with symptom burden (*β* = -0.01, *t* = -0.179, *p* = 0.858). Similarly, at low mindfulness levels, demoralization increased sharply with higher levels of avoidance coping (*β* = 0.29, *t* = 7.15, *p* < 0.001). At high mindfulness levels, although demoralization still increased with avoidant coping, the slope was substantially attenuated (*β* = 0.14, *t* = 2.99, *p* = 0.003).

**Figure S1.** Simple slope diagram of the moderating effect of trait mindfulness.

**Multimedia Appendix 1-5**

The researcher conducted in-depth interviews with 14 MHD patients and finally summarized 4 themes and 16 sub-themes of psychological experience of demoralization, impediments to improvement of demoralization, facilitators of improvement of demoralization, and needs for intervention (Table S7).

**Table S7.** Qualitative Research Themes on Learned Helplessness Syndrome in MHD Patients

| Thematic | Sub-thematic |
| --- | --- |
| Theme 1: Psychological Experience of demoralization | meaninglessness |
|  | Decreased sense of personal worth |
|  | Desperate Experience |
|  | uncertainty about the future |
| Theme 2: Impediments to Improvement of demoralization | Stigma and social isolation |
|  | Symptom burden versus emotional burden |
|  | avoidance action |
|  | Lifestyle changes |
| Theme 3: Facilitators of Improvement in demoralization | Family support |
|  | social security |
|  | Peer Support |
|  | medical coping styles -confront |
| Theme 4: Demand for intervention | Flexible scheduling |
|  | Easy to understand content |
|  | Format Interactive Feedback |
|  | With incentives |

***Theme 1: Psychological Experience of Demoralization***

**Meaninglessness**

During the interviews, several patients expressed a sense of meaninglessness in life. MHD patients face significant life changes and physical challenges after dialysis that provoke them to question the meaning of life. *At that time, the heart is just very stressful, just don't know whether today or tomorrow will be saying goodbye to the world, this person is living too meaningless (N4). Now I always feel resigned, why is it like this ...... I feel that life is meaningless(N12).*

**Decreased sense of personal worth**

Some patients suffered from impaired self-esteem related to changes in occupational roles and reduced physical abilities, which deprived them of the sense of accomplishment and satisfaction they gained from their labor, leading to a reduced sense of personal worth. *I feel useless, a liability, and a nuisance to my family(N9).*

**Desperate Experience**

The despair and helplessness that most patients feel when they enter MHD treatment is filled with unease and fear about life and the future. *Anyway, my mood after I got the disease, it was always quite desperate(N2). At that time, it felt like the sky was falling, it felt like it was impossible to live, like life was almost over(N6).*

**Uncertainty about the future**

Uncertainty about the future is common among MHD patients. This uncertainty is primarily characterized by concerns about physical functioning and fear of unforeseen events that may occur during treatment. *When I'm not feeling well I wonder if I'm going to fail, right away. It would be like that a lot, when I'm not feeling well, I'd be thinking about it(N5). In dialysis, we patients have to resuscitate. Other patients are still on dialysis one day, and the next day family members in the group post obituaries, and it feels especially heavy. I'm a little worried that I don't know when this kind of thing will happen to myself(N7).*

***Theme 2: Impediments to Improvement in the demoralization***

**Stigma and social isolation**

MHD patients are socially isolated due to the stigma associated with the disease, which makes them feel different from others. *You always go out into society feeling different from people as well(N2).* Stigma and the need for dialysis treatment led to significant changes in the lifestyles of most patients, not only limiting their social activities but also affecting their interactions with family members, creating significant social barriers and enhancing their sense of isolation. *It doesn't particularly affect my work, but I can't participate in so many activities(N7). I used to like talking to my neighbors, but now, I don't even really want to meet people. I'm afraid they'll see me like this and think I'm sick(N10).* Some patients felt that they did not have the understanding or support of others and did not want to bother others. Social isolation was exacerbated by limitations on social activities. *I wasn't interested in anything when I was sick and I didn't want to talk to others. I felt no one could understand me to help me and I didn't want to bother others(N9).*

**Symptom burden versus emotional burden**

Most patients suffer from a multifaceted symptom burden, including itching, fatigue, insomnia, and pain, which has an impact on their psychological and physical health, as well as on the quality of their daily lives. *The skin is very itchy and it always feels uncomfortable(N2). After I had blood dialysis, I personally felt that my reaction was a little bit slow and I couldn't sleep anymore. There is a little deformation of the bones, and it is especially painful to land on the heel, and I can't walk(N4).*

Emotional burdens from fellow patients: MHD patients are affected by emotional burdens from fellow patients, which exacerbate their anxiety, fear, and negatively affect the psyche. *Some of us sick people who are older or whatever sudden situation, old bad (deaths), inevitably we think about ourselves, and over time, that's chronic anxiety. It's like thinking about whether you're going to be gone one day just like that(N8).*

Emotional burden from family: MHD patients often consider the feelings and expectations of their family members as they face dialysis. Family concern and expectations, while an emotional support, can be a source of stress for the patient. *After all, it's a serious illness, say in case of any accidents or anything, my biggest fear is to drag my family down(N6). Parents always want their children to be better. My father is also very sad about this disease, but he can't do much to help(N2).*

**Avoidance action**

Some patients adopt avoidance strategies, choosing to conceal their illnesses to avoid social and public pressure. In daily social situations, they are also reluctant to be labeled as “patients” and are ostracized from social life. *Many of us here, including my relatives, don't know that I am a dialysis patient, and I don't talk much about it. I don't want to hear the word “uremia” (N10). Anyway, my relatives and friends don't know that I have this disease, I hide it from them. There must be some concerns, and the village must look at it as a joke (with tears), because people in the countryside must talk about you when they know you have the disease(N11). Other people have reunions at Chinese New Year, visiting friends and having parties. I'm a sick person, and I feel like I have the word 'sick' hanging over my head(N7).*

**Lifestyle changes**

Dialysis time constraints: Most patients reported that the duration and frequency of dialysis interfered with their daily lives. *For us, it felt like dialysis was going to be three trips to the hospital every week, and it was like being fixed in the hospital, and there was no freedom(N6).* Employment limitations: MHD has had an impact on the careers of some patients, with physical limitations due to the disease preventing them from carrying out their regular occupational activities. *Now that I have the disease, I would have liked to run a drip, it seems like I don't have the stamina, a little bit of stamina to keep up(N11). Dialysis first of all has an impact on work, basically overwhelmed on top of work(N6).* Travel restrictions: Some patients indicated that they were unable to arrange long-distance travel due to the need for regular dialysis, and even if they were able to find a dialysis hospital, the related preparations such as blood tests would cause trouble and distress. *Can't go out and travel far, only close by. The impact is definitely a little bit(N11). Can't travel either, and it's troublesome to find a designated dialysis hospital when you travel, and you have to take blood tests and stuff, so it's too much trouble(N7).* Dietary restrictions: some patients reported that hemodialysis restrictions on eating and drinking also affect their social interactions and psychological state. *I can't eat anything; I always feel a little bit sorry for it. Yesterday I went to have a wedding drink, people kept shouting at me to eat, and after I ate I felt how it was so sinful(N1). You can't eat, you can't eat, you can't eat without nutrients, and dialysis will give him more strength, and you can't eat too much(N10).*

***Theme 3: Facilitators of improvement in demoralization***

**Family support**

Most patients reported that family love and support played a positive role in their mental health and ability to cope in the face of dialysis treatment. *My family is exceptionally good, they are all very supportive, encouraging, and caring(N3). My two sisters, two brothers. Every week they come and visit and help me to look at me(N4).*

**Social security**

Some MHD patients receive assistance and care from various aspects of society, such as medical personnel, work units and the government. The multifaceted support not only meets the patients' financial and material needs, but also provides them with emotional comfort and psychological support, which helps them better adapt to the treatment process and maintain a positive mindset, thus improving their quality of life. *The school is still taking better care of me, for one thing, I am about to retire, and for another, I am suffering from a major disease. There is also an economic aspect, the Party and the government have given us a lot of help, in this regard we should thank the Party and the government(N6).*

**Peer Support**

Peer support demonstrates its indispensable role. Patients' mutual communication and sharing not only provide valuable experience, but also bring psychological comfort and courage. Through experience sharing, emotional communication and common activities among patients, patients were able to gain emotional comfort, psychological support and courage in life, which not only helped them better adapt to the dialysis treatment, but also significantly improved their quality of life. *Because we have frequent communication among our patients. And then we have old patients there who have been doing it for more than ten years and they all pass on their experience(N1). One of us who has been on dialysis for more than 20 years, he is the light of enlightenment in my life, I feel like I'm going to be unable to live without him(N12).*

**Meet head-on**

Some patients adopted positive coping strategies, including facing life positively, accepting the reality of the disease, and seeking psychological support. *I was thinking that it would be nice to have a counselor or something for our blood dialysis piece. It would be nice. I really thought about it that way(N8). Hemodialysis is like going to work and going off work, and when you're done, you come back from work and go to bed(N4).*

***Theme 4: Demand for intervention***

**Flexible scheduling**

MHD patients' need for psychological interventions is reflected in scheduling flexibility. They tend to prefer interventions that can be conducted at home and are not restricted by time and place. This flexibility not only helps patients to better manage their health, but also improves their motivation and continuity in participating in the intervention. *I don't know much about online positive thinking, but I am quite interested. If it can be practiced right at home, I am willing to try it(N13). I usually have to work and have blood dialysis, I hope that these exercises are more flexible and can be done anytime and anywhere so that they won't affect my work and dialysis(N6). We do hemodialysis and can't go out often, so it's better to practice at home(N7).*

**Easy to understand content**

Some MHD patients felt that the content of psychological interventions needed to be easy to understand. They tended to choose simple, intuitive, and easy-to-understand content to learn and practice on their own at home. *For the practice of online positive thinking, it is better to be simple and easy to follow without much extra time and effort(N1). Don't be too complicated with these exercises of yours. I'm an older person, so if there are videos or pictures or something like that, it might be easier(N10). I don't know much about what you are talking about. However, if someone can teach me, I am willing to try. After all, I can't go out far now, so it would be good for me to learn something at home. I'm just afraid that this internet stuff is too complicated for me to learn. Because I don't read much, it's too complicated that I might not be able to learn it(N14).*

**Format Interactive Feedback**

Some MHD patients believe that psychological interventions should have interaction and feedback, and they tend to choose interventions that can provide social interaction, group support, and professional guidance. *It is better to have some interactive sessions in the practice, such as having professional guidance or communicating with other participants, so it is easier to stick to it(N7). In the WeChat group, we can also communicate with each other, what do you feel when you practice? How do I feel after practicing? This will only be more effective after communication, it's hard for one person to stick to it because people influence each other(N8).*

**With incentives**

Patients have different levels of education and receptivity to new knowledge, and interventions should include more individualized and diversified motivational strategies to meet the needs of patients with different cultural and educational backgrounds. *Then you for example I can create an incentive mechanism from the perspective of mechanism building. For example, it was our hospital that got it pretty good and built a Microsoft program and gave me top priority(N12).*

**Multimedia Appendix 1-6**

***Table S8****.* *Demographic and clinical disease information of the two groups and comparison (N=72*)

| Variable | intervention group(*N*=36) | control group(*N*=36) | *χ2(df)* | *p* |
| --- | --- | --- | --- | --- |
| Gender |  |  |  |  |
| Male | 20(55.56%) | 16(44.44%) | 0.89(1) | 0.346 |
| Female | 16(44.44%) | 20(55.56%) |  |  |
| Age |  |  |  |  |
| 18-40 year | 3(8.33%) | 4(11.11%) | 2.82(3) | 0.431 |
| 41-50 year | 10(27.78%) | 6(16.67%) |  |  |
| 51-60 year | 12(33.33%) | 18(50.00%) |  |  |
| 61-65year | 10(30.56%) | 8(22.22%) |  |  |
| Education level |  |  |  |  |
| Junior high school or below | 31(86.11%) | 25(69.44%) | 3.56(2) | 0.168 |
| Senior high school or technical secondary school | 2(5.56%) | 7(19.44%) |  |  |
| College and above | 3(8.33%) | 4(11.11%) |  |  |
| Occupational status |  |  |  |  |
| out of work | 10(27.78%) | 10(27.78%) | 0.103(2) | 0.950 |
| retirement | 20(55.56%) | 19(52.78%) |  |  |
| incumbency | 6(16.67%) | 7(19.44%) |  |  |
| Marital status |  |  |  |  |
| Married | 31(86.11%) | 30(83.33%) | 0.216(2) | 0.897 |
| Unmarried | 3(8.33%) | 3(8.33%) |  |  |
| Divorced or widowed | 2(5.65%) | 3(8.33%) |  |  |
| Religious belief |  |  |  |  |
| Yes | 22(61.11%) | 25(69.44%) | 0.551(1) | 0.458 |
| No | 14(38.89%) | 11(30.56%) |  |  |
| Residency |  |  |  |  |
| With the family | 34(94.44%) | 35(97.22%) | 1.014(2) | 0.602 |
| Live alone | 1(2.78%) | 1(2.78%) |  |  |
| The rest | 1(2.78%) | 0(0.00%) |  |  |
| current address |  |  |  |  |
| Countryside | 15(41.67%) | 12(33.33%) | 1.173(2) | 0.556 |
| Cities and towns | 13(36.11%) | 12(33.33%) |  |  |
| Municipalities | 8(22.22%) | 12(33.33%) |  |  |
| Medical payment methods |  |  |  |  |
| Medical insurance | 34(94.44%) | 33(91.67%) | 0.215(1) | 0.643 |
| Farmer’s insurance | 2(5.56%) | 3(8.33%) |  |  |
| Disease-induced burdens |  |  |  |  |
| Very heavy | 12(33.33%) | 16(44.44%) | 2.153(2) | 0.341 |
| General | 24(66.67%) | 19(52.78%) |  |  |
| Light | 0(0.00%) | 1(2.78%) |  |  |
| Self-care ability |  |  |  |  |
| Self-care | 32(88.89%) | 32(88.89%) | 0.533(2) | 0.766 |
| Partially self-care | 3(8.33%) | 2(5.56%) |  |  |
| Unable self-care | 1(2.78%) | 2(5.56%) |  |  |
| Primary disease |  |  |  |  |
| DN | 2(5.56%) | 7(19.44%) | 4.93(3) | 0.177 |
| HN | 14(38.89%) | 11(30.56%) |  |  |
| CG | 8(22.22%) | 11(30.56%) |  |  |
| the rest | 12(33.33%) | 7(19.44%) |  |  |
| Dialysis age |  |  |  |  |
| ＜1year | 5(13.89%) | 3(8.33%) | 2.90(2) | 0.234 |
| 1-5year | 11(30.56%) | 18(50.00%) |  |  |
| ＞5year | 20(55.56%) | 15(41.67%) |  |  |
| Frequency of dialysis |  |  |  |  |
| 3 times a week | 35(97.22%) | 35(97.22%) | 0.00(1) | 1.000 |
| 2 times a week | 1(2.78%) | 1(2.78%) |  |  |
| Type of medication |  |  |  |  |
| 1-3 species | 13(36.11%) | 14(38.89%) | 0.78(2) | 0.677 |
| 4-7 species | 20(55.56%) | 17(47.22%) |  |  |
| 7 or more | 3(8.33%) | 5(13.89%) |  |  |

***Table S9.*** *Status and comparison of each study variable in the two groups before the intervention (N=72*)

| Variable | Intervention Group (*n*=36) | Control Group (*n*=36) | Mean Difference | *p* | Effect Size  （Cohen’s *d*, 95%CI） |
| --- | --- | --- | --- | --- | --- |
| **Demoralization** | | |  |  |  |
| Baseline | 16.42 ± 3.97 | 16.75 ± 4.21 | -0.333 | 0.731 | 0.002 (-2.26, 1.59) |
| Week 6 | 11.33 ± 3.61 | 16.39 ± 4.29 | -5.063 | < 0.001 | 0.296 (-6.92, -3.20) |
| **Trait Mindfulness** | | |  |  |  |
| Baseline | 38.47 ± 7.54 | 36.72 ± 5.84 | 1.750 | 0.275 | 0.017 (-1.42, 4.29) |
| Week 6 | 43.54 ± 3.65 | 37.06 ± 4.13 | 6.480 | <0.001 | 0.416 (4.65, 8.31) |
| **Confrontation Coping** | | |  |  |  |
| Baseline | 17.19 ± 2.64 | 17.17 ± 2.68 | 0.028 | 0.965 | 0.000 (-1.22, 1.28) |
| Week 6 | 23.75 ± 2.05 | 17.86 ± 2.24 | 5.886 | <0.001 | 0.658 (4.88, 6.90) |
| **Avoidance Coping** | | |  |  |  |
| Baseline | 16.47 ± 3.02 | 17.17 ± 2.24 | -0.694 | 0.271 | 0.017 (-1.94, 0.55) |
| Week 6 | 12.58 ± 2.37 | 17.42 ± 1.70 | -4.841 | <0.001 | 0.587 (-5.81, -3.87) |
| **Resignation Coping** | | |  |  |  |
| Baseline | 12.94 ± 2.68 | 13.44 ± 1.78 | -0.500 | 0.355 | 0.012 (-1.57, 0.57) |
| Week 6 | 12.56 ± 3.24 | 13.89 ± 1.67 | -1.328 | 0.032 | 0.064 (-2.54, -0.12) |
| **Symptom Burden** | | |  |  |  |
| Baseline | 150.17 ± 52.11 | 162.78 ± 31.69 | -12.611 | 0.22 | 0.022 (-32.88,7.66) |
| Week 6 | 132.90 ± 44.40 | 162.06 ± 29.82 | -29.153 | 0.002 | 0.133(-46.93, -11.37) |

**Table S10.** *Sensitivity analysis (n = 68).*

| Variable | intervention group  (*n*=32) | control group  (*n*=36) | Mean Difference | *p* | Effect Size  （Cohen’s *d*, 95%CI） |
| --- | --- | --- | --- | --- | --- |
| **Demoralization** | | |  |  |  |
| Baseline | 16.72 ± 4.10 | 16.75 ± 4.21 | -0.031 | 0.975 | 0.000 (-2.05, 1.99) |
| Week 6 | 11.03 ± 3.61 | 16.39 ± 4.29 | -5.063 | <0.001 | 0.317 (-7.29, -3.43) |
| **Trait Mindfulness** | | |  |  |  |
| Baseline | 38.28 ± 7.67 | 36.72 ± 5.84 | 1.559 | 0.346 | 0.013 (-1.72, 4.84) |
| Week 6 | 43.69 ± 3.69 | 37.06 ± 4.13 | 6.632 | <0.001 | 0.422 (4.73, 8.54) |
| **Confrontation Coping** | | |  |  |  |
| Baseline | 17.22 ± 2.79 | 17.17 ± 2.68 | 0.052 | 0.965 | 0.000 (-1.27, 1.38) |
| Week 6 | 24.22 ± 1.60 | 18.19 ± 2.36 | 6.024 | <0.001 | 0.691 (5.03, 7.02) |
| **Avoidance Coping** | | |  |  |  |
| Baseline | 16.50 ± 3.01 | 17.17 ± 2.24 | -0.667 | 0.30 | 0.016 (-1.94, 0.61) |
| Week 6 | 12.22 ± 2.06 | 17.42 ± 1.70 | -5.198 | <0.001 | 0.663 (-6.11,-4.29) |
| **Resignation Coping** | | |  |  |  |
| Baseline | 12.91 ± 2.84 | 13.44 ± 1.78 | -0.538 | 0.347 | 0.013 (-1.67, 0.60) |
| Week 6 | 9.41 ± 2.27 | 13.89 ± 1.67 | -4.483 | <0.001 | 0.57 (-5.44, -3.53) |
| **Symptom Burden** | | |  |  |  |
| Baseline | 150.41 ± 54.05 | 162.78 ± 31.69 | -9.37 | 0.38 | 0.012 (-30.54, 11.80) |
| Week 6 | 133.97 ± 46.16 | 162.0 ± 29.82 | -28.09 | 0.004 | 0.121 (-46.70, -9.47) |
